# Alternative splicing in voltage-gated sodium channels: mechanisms, regulatory networks and therapeutic implications

**DOI:** 10.1080/07853890.2026.2645735

**Published:** 2026-03-23

**Authors:** Jiaying Qiu, Pei Wu, Yalin Zhang, Yiqing Li, Chunli Xia, Siwan Peng, Junjie Sun

**Affiliations:** a Key Laboratory of Neuroregeneration of Ministry of Education, Co-innovation Center of Neuroregeneration, Jiangsu Key Laboratory of Tissue Engineering and Neuroregeneration, Nantong University, Nantong, PR China; b Department of Prenatal Screening and Diagnosis Center, Nantong Key Laboratory of Prenatal Diagnosis, Affiliated Maternity and Child Health Care Hospital of Nantong University, Nantong, PR China; c Department of Gynecology, Nantong Maternal and Child Health Hospital of Nantong University, Nantong, PR China; d Life Sciences School of Nantong University, Nantong, PR China

**Keywords:** VGSCs, alternative splicing, RNA-binding proteins, precision medicine, spliceopathy

## Abstract

**Background:**

Voltage-gated sodium channels (VGSCs) are fundamental to electrical signalling in excitable cells, and their dysfunction underlies a wide range of channelopathies. While the existence of nine distinct α-subunit genes contributes to VGSC diversity, alternative splicing serves as a significant post-transcriptional mechanism that profoundly expands their proteomic and functional repertoire. Dysregulation of this splicing process is increasingly linked to disease pathogenesis.

**Introduction:**

This review aims to provide a comprehensive synthesis of the alternative splicing landscape across all nine VGSC α-subunits. It systematically catalogs known splicing variants, details their roles in developmental regulation, tissue-specific expression and fine-tuning of channel biophysics, and examines the regulatory networks controlling these events.

**Discussion:**

We detail conserved splicing switches (e.g. the 5N/5A exon in neuronal channels) and isoform-specific events across the VGSC family (Nav1.1 to Nav1.9), evaluating their functional and clinical impacts. The regulation of these events by key RNA-binding proteins (RBPs), such as Rbfox and Nova2, within cell-type-specific networks is emphasized. Furthermore, we discuss how splicing dysregulation contributes to channelopathies and evaluate the promising potential of novel therapeutic strategies, particularly antisense oligonucleotides (ASOs), to correct pathogenic splicing defects.

**Conclusions:**

By integrating mechanistic insights with clinical implications, this review establishes alternative splicing as a central theme in VGSC biology and pathophysiology. It highlights the critical need for, and the emerging path towards, precision medicine approaches that target splicing defects for the treatment of VGSC-associated disorders, providing a foundational resource to guide future research and therapeutic development.

## Key messages

- Alternative splicing critically expands the functional diversity of voltage-gated sodium channels (VGSCs).
- Dysregulation of this splicing process is a direct cause of disease, contributing to various channelopathies.
- Correcting pathogenic splicing defects, notably via antisense oligonucleotides (ASOs), represents a promising precision therapeutic strategy.

## 1. Introduction

Electrical signalling constitutes the fundamental mechanism for communication in the nervous system, muscle contraction and cardiac pacemaking. This process depends on precisely regulated ionic fluxes across the plasma membrane through ion channels. By acting as gatekeepers of ionic movement, ion channels confer electrical excitability to cells and govern physiological processes ranging from neurotransmitter release to gene expression [1–3]. Consequently, ion channel dysfunction – due to inherited or acquired mutations – leads to a spectrum of disorders known as channelopathies, including epilepsy, cardiac arrhythmias, myotonias and neuropathic pain [4–7].

Among ion channel families, voltage-gated sodium channels (VGSCs) are critical for initiating and propagating action potentials in excitable cells [8]. Humans have nine types of VGSCs, namely Nav1.1–Nav1.9, which are encoded by *SCN1A*–*SCN5A* and *SCN7A*–*SCN11A* [9]. Each VGSC functions as a macromolecular complex containing a pore-forming α-subunit and one or more auxiliary β-subunits. The α-subunit determines core channel properties, whereas β-subunits modulate gating kinetics and voltage dependence [10].

**Figure 1.**
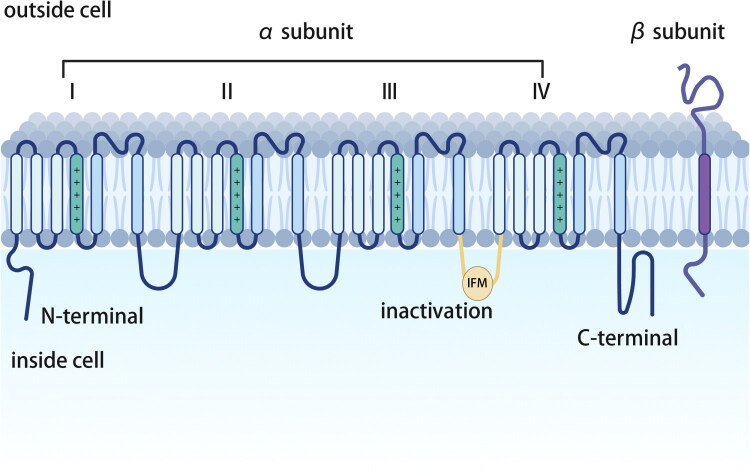
Schematic structure of the voltage-gated sodium channel (VGSC) α-subunit and its association with an auxiliary β-subunit.

Structurally, the α-subunit consists of four homologous domains (D1–D4), each containing six transmembrane segments (S1–S6). The isoforms share high homology, with >50% amino acid conservation in transmembrane and extracellular domains [11]. The S5–S6 linker forms the pore loop, and the positively charged S4 segments serve as voltage sensors; depolarization triggers their outward movement, leading to channel activation [12]. β-Subunits are 33–36 kDa single-pass transmembrane proteins that associate non-covalently or via disulphide bonds with the α-subunit. They also interact with cell adhesion molecules and extracellular matrix (ECM) proteins, contributing to channel localization, cell adhesion and migration (Figure 1) [10,13–15]. In recent years, near-atomic-resolution cryo-EM structures have been determined for multiple eukaryote VGSC isoforms, including Nav1.1, Nav1.2, Nav1.5, Nav1.6, Nav1.7 and Nav1.9. These structures provide unprecedented atomic-level insights into the pore architecture, voltage-sensing conformations, auxiliary subunit interfaces and state-dependent modulation mechanisms [16–18]. A notable example is the structure of human Nav1.6, which revealed a novel, structurally unresolved binding pocket within the pore domain, offering a potential target for developing subtype-specific therapeutics [19]. Collectively, this structural revolution has fundamentally transformed our understanding of VGSC function, disease pathogenesis and drug discovery, opening new avenues for precise mechanistic and translational research.

The canonical VGSC is depicted as a transmembrane complex. The pore-forming α-subunit consists of four homologous domains (DI–DIV), each containing six transmembrane segments (S1–S6). The S4 segment in each domain (shown in red) serves as the voltage sensor. The extracellular loops between S5 and S6 (pore loops, P-loops) form the ion selectivity filter. The short intracellular linker between DIII and DIV harbors the critical isoleucine–phenylalanine–methionine (IFM) motif, which acts as the fast inactivation gate. The intracellular N- and C-terminal are involved in channel modulation and trafficking. A single transmembrane β-subunit is shown non-covalently associated with the α-subunit, modulating channel gating, trafficking and cell adhesion. This schematic illustrates the foundational architecture underlying the functional diversity achieved through alternative splicing, as discussed in the review.

Alternative splicing of VGSCs is crucial because it generates multiple functionally distinct subtypes from a single gene, greatly enriching the diversity of electrical signal regulation and thus meeting the specific physiological needs of different tissues (such as nerves and muscles) and developmental stages. Abnormal splicing patterns of VGSCs disrupt sodium current balance, directly leading to various diseases such as epilepsy, pain and arrhythmias [20,21]. Furthermore, abnormal VGSC expression and splicing also play an important role in various non-excitable tissue diseases, especially cancer. Multiple tumour-specific splicing isoforms of VGSCs are directly related to malignant phenotypes such as tumour invasion, metastasis, proliferation and drug resistance [22]. Therefore, alternative splicing of VGSCs not only represents the molecular basis of physiological complexity but also helps to elucidate the pathogenesis of diseases (epilepsy, cancer, etc.), laying the foundation for further precise drug treatment [20,23].

This review aims to fill this gap by comprehensively summarizing the alternative splicing profiles of various VGSC subtypes (Nav1.1–Nav1.9), analysing their functional impact and regulatory mechanisms, and paying particular attention to the role of splicing variants in the progression of certain diseases. We will also explore potential therapeutic strategies targeting pathogenic splicing events, hoping to provide new ideas and resources for the research and treatment of VGSC-related diseases.

## 2. The mechanisms of alternative splicing

**Figure 2.**
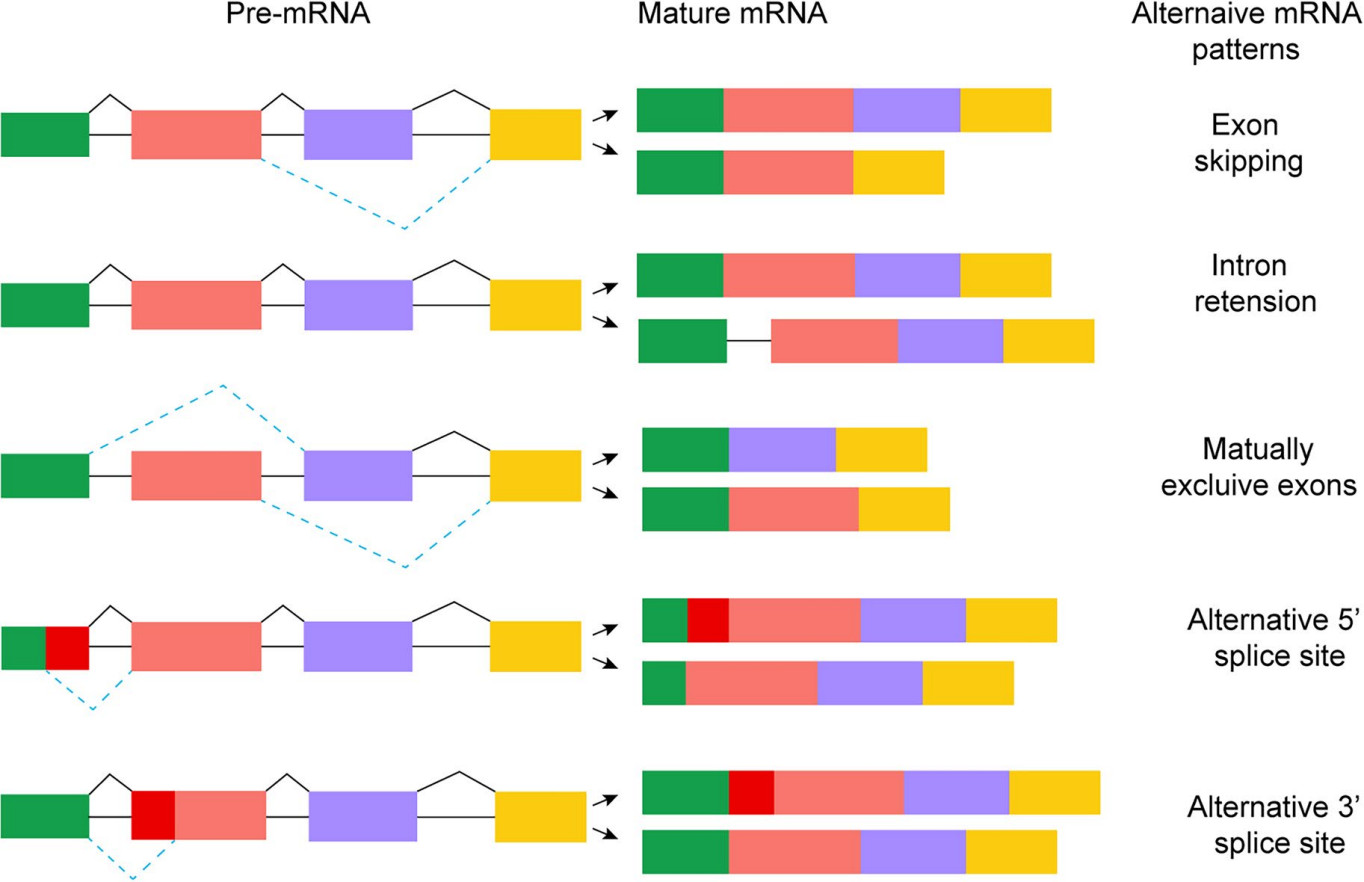
Major patterns of alternative splicing in eukaryotic pre-mRNA.

Alternative splicing of pre-messenger RNA (pre-mRNA) allows a single gene to produce multiple protein isoforms with diverse structures and functions, significantly expanding the complexity of genome expression and the adaptability of organisms [24]. Alternative splicing events are mainly classified into five modes: exon skipping, intron retention, mutually exclusive exons, alternative 5′ splice site selection and alternative 3′ splice site selection (Figure 2). In VGSC-encoding genes, reported forms of alternative splicing include exon skipping, mutually exclusive exons, and alternative 5′ and 3′ splice site.

Alternative splicing is a key post-transcriptional mechanism that expands transcriptomic and proteomic diversity from a single gene. This schematic illustrates five principal patterns: *Exon skipping* (cassette exon): An exon is either included in or excluded from the mature mRNA. *Mutually exclusive exons*: Only one of two (or more) adjacent exons is retained in the final transcript. *Alternative 5′ splice site selection*: The use of different donor splice sites alters the 5′ boundary and length of an exon. *Alternative 3′ splice site selection*: The use of different acceptor splice sites alters the 3′ boundary and length of an exon. *Intron retention*: An intron remains unspliced and is retained in the mature mRNA. Exons are shown in multiple colours and introns as lines.

Alternative splicing is regulated by a sophisticated system, the core components of which are cis-acting elements and trans-acting factors. Cis-acting elements refer to motifs composed of specific sequences located on the pre-mRNA, with 5′ and 3′ splice sites, branch point sequences and polypyrimidine tracts being core signals. These sequences are usually located at specific sites in introns, are highly conserved, and directly determine whether splicing can proceed. In addition, there are some cis-regulatory elements with more flexible positions and functions, including splicing enhancers (exonic splicing enhancer (ESE), intronic splicing enhancer (ISE)) and splicing silencers (exonic splicing silencer (ESS), intronic splicing silencer (ISS)), which participate in regulating the extent of alternative splicing. Trans-acting factors are mainly RNA-binding proteins (RBPs) that regulate splicing by recognizing and binding to cis-acting elements, and are therefore also called splicing regulatory factors. For example, Rbfox protein binds to pre-mRNA by recognizing the conserved ‘(U)GCAUG’ motif, thereby increasing the proportion of the *SCN8A* gene 18A splicing isoform [25]. More than 3000 proteins with RNA-binding capabilities have been identified in mammalian cells; however, the high-affinity binding motifs of most of these proteins and their specific roles in alternative splicing remain unclear, leaving a large number of ‘splicing codes’ yet to be deciphered [26].

Splicing regulatory factors mainly regulate alternative splicing at three levels. The first level is expression-dependent regulation, where changes in the expression level of a single regulatory factor affect its binding to pre-mRNA, thereby causing changes in alternative splicing. For example, the Rbfox protein is crucial for brain development; knocking out this protein alters the alternative splicing pattern of *Scn8a* and leads to neuronal electrophysiological dysfunction [27]. The second layer of regulation is position-dependent regulation, where the function of certain RBPs is independent of their expression levels but depends on their binding location on the pre-mRNA. For instance, hnRNPA1/A2 (heterogeneous nuclear ribonucleoproteins) exhibits strong splicing inhibitory function when located near the 5′ splice site, but switches to promoting splicing when bound near the 3′ splice site [28]. In a real cellular environment, numerous RBPs coexist and interact in the same spatiotemporal context, constituting the third layer of regulation – context-dependent regulation. Different RBPs can bind to the same or different locations on the same pre-mRNA, and collectively determine the alternative splicing pattern of the target gene through cooperative or antagonistic effects. Due to limitations of current research methods, it is difficult to capture all RBPs that bind to a specific pre-mRNA in a high-throughput and real-time manner. Therefore, research on alternative splicing regulation, including that of the VGSC gene, still mainly relies on cell lines or cell-free systems, and only a few RBPs have had their functions elucidated. The complete picture of context-dependent regulation remains difficult to fully reveal.

## 3. Voltage-gated sodium channel splice variants: functional diversification and pathophysiological implications

The functional diversity of VGSCs extends beyond the existence of nine distinct α-subunit genes, being substantially amplified by alternative splicing. This key post-transcriptional mechanism generates a wide array of functionally specialized isoforms from individual genes, enabling precise adaptation of channel properties to tissue-specific requirements, developmental stages and physiological contexts. This section systematically reviews documented alternative splicing events across the VGSC family, focusing on their biophysical consequences, regulatory mechanisms and emerging roles in disease pathogenesis. Major splicing events are summarized in Table 1.

**Table 1.** Major splicing events of VGSCs.

Nav isoform	Gene	Alternative splicing event	Splicing type	Key functional/clinical impact	Regulatory factors
Nav1.1	*SCN1A*	Exon 5A (adult) vs. 5N (neonatal)	Mutually exclusive	Alters drug response; 5N linked to febrile seizures and carbamazepine resistance	Nova2, SCNM1
Nav1.2	*SCN2A*	Exon 6A vs. 6N	Mutually exclusive	5N has depolarized activation voltage; may protect neonatal brain from hyperexcitability	(unknown)
Nav1.3	*SCN3A*	Exon 6A vs. 6N Exon 12 (12v1, v2, v3, v4)	Cassette exon	12v2 subtype exhibits depolarization shift with steady-state inactivation	(Potential Nova2)
Nav1.5	*SCN5A*	Multiple (e.g. △exon18-Nav1.5a; exon 6A vs. 6N)	Exon skipping, mutually exclusive	Alters inactivation kinetics, current density; neonatal isoform promotes invasiveness in cancer	(unknown)
Nav1.6	*SCN8A*	Exon 5A vs. 5N; exon 18A vs. 18N	Mutually exclusive	18A inclusion is essential for functional channels; regulated developmentally and by Rbfox	Rbfox1/2, SCNM1
Nav1.7	*SCN9A*	Exon 6A (adult) vs. 6N (neonatal); exon 12L vs. 12S	Mutually exclusive, cassette exon	6A slows inactivation; 12S is modulated by PKA, implicated in pain after nerve injury.	(unknown)
Nav1.8	*SCN10A*	NAGNAG site (Q1030 in/out)	Alternative 3′SS	The biophysical influence is weak in humans; evolutionary significance to be elucidated	(unknown)
Nav1.9	*SCN11A*	E23c inclusion (Nav1.9b)	Cassette exon (intron retention)	Nav1.9b is a putative truncated protein; function unknown.	(unknown)

### 3.1. Developmental splicing switches in central nervous system sodium channels: Nav1.1, Nav1.2 and Nav1.6

A conserved alternative splicing mechanism that modifies the S3–S4 linker in domain I represents a hallmark of several neuronal VGSCs. This process involves mutually exclusive exons – designated 5N (neonatal) and 5A (adult) – and constitutes a developmentally programmed splicing switch.

In *SCN1A* (Nav1.1), splicing of exons 5A and 5N holds significant clinical relevance. A common intronic polymorphism (IVS5-91 G > A) disrupts the 5′ splice site of exon 5N, promoting its preferential exclusion and leading to predominant expression of the 5A-containing transcript in the adult brain [29]. Although the mouse ortholog lacks a functional 5N exon, reduced inclusion of exon 5N in humans is associated with an elevated risk of febrile seizures in children and with carbamazepine resistance in epilepsy patients [29–31]. These findings indicate that the neonatal Nav1.1 isoform, while not essential for development, plays a key modulatory role in neuronal circuit stability, and its altered expression can lower the seizure threshold.

In *SCN2A* (Nav1.2), the Nav1.2-N (5N) and Nav1.2-A (5A) splice variants differ by a single amino acid substitution (D209N). Functionally, the neonatal Nav1.2-N variant exhibits a depolarizing shift in the voltage dependence of activation compared to the adult Nav1.2-A isoform [32,33], indicating that the neonatal channel requires stronger depolarization to open. This may act as an intrinsic brake on neuronal excitability during early brain development. Notably, several early-onset epileptic encephalopathy-causing mutations in *SCN2A* exert more severe effects on the neonatal Nav1.2-N variant than on the adult isoform [34]. This provides a molecular rationale for the clinical improvement observed in some patients as their brains mature and transition towards the adult splice variant, underscoring the therapeutic importance of understanding developmental splicing regulation.

The *SCN8A* (Nav1.6) gene undergoes two distinct layers of alternative splicing, contributing to its functional complexity. In addition to the conserved 5N/5A switch in domain I, it contains another pair of mutually exclusive exons, 18N and 18A, in domain III. Inclusion of exon 18A is critical because it encodes the complete S3–S4 voltage-sensing segment, whereas exon 18N harbors a premature termination codon, leading to nonsense-mediated decay (NMD) of the transcript and preventing production of full-length functional channels [25,35]. The developmental transition from embryonic 5N/18N transcripts to adult 5A/18A transcripts in the brain is tightly regulated by neuronal splicing regulators, particularly the Rbfox family of RBPs. Knockdown of Rbfox1 and Rbfox2 in mice significantly reduces inclusion of exon 5A and, more profoundly, exon 18A, resulting in decreased Nav1.6 protein levels and abnormal pacemaking activity in Purkinje neurons [25,27]. Nav1.6 splice variants are significantly associated with human diseases. This connection is primarily evidenced in two key aspects. First, loss-of-function mutations or copy number variations in RBFOX1 – a core splicing regulator – have been linked to human intellectual disability and autism spectrum disorders. The pathogenic mechanism is partly attributed to its disruption of the splicing of downstream target genes such as *SCN8A*, leading to Nav1.6 dysfunction. Second, the critical exon 18A of *SCN8A* undergoes a developmentally regulated splicing switch: it does not become the overwhelmingly dominant isoform until around 10 months of age in infancy. This crucial transition period closely coincides with the onset age of many SCN8A-related neurodevelopmental disorders, such as developmental and epileptic encephalopathies [25]. Consequently, disruption of the RBFOX-mediated regulatory program that ensures correct expression of the adult splice variant may impede this essential step in nervous system maturation, thereby contributing to disease pathogenesis.

### 3.2. Nav1.3: splice variants in the I–II linker with subtle biophysical tuning

In contrast to the mutually exclusive exon switches in domain I of other neuronal sodium channels, alternative splicing in Nav1.3 centres on exon 12, which encodes part of the intracellular linker between domains I and II – a region involved in channel modulation. This process generates four principal isoforms (designated 12v1–12v4) [36]. In humans, the 12v1 isoform predominates, while 12v4 is expressed at markedly low levels. Biophysical characterization in heterologous systems reveals that these splice variants form functional channels with only minor differences in tetrodotoxin (TTX) sensitivity and activation/deactivation kinetics [37]. A more notable distinction is observed in steady-state inactivation: the 12v2 isoform exhibits a depolarizing shift in its voltage dependence of inactivation compared to the 12v4 variant. This suggests a potential cell-type-specific mechanism for fine-tuning channel availability and neuronal excitability, the full physiological relevance of which awaits further elucidation *in vivo*.

### 3.3. Nav1.5: a spectrum of splice variants with cardiac and non-cardiac roles

The cardiac sodium channel Nav1.5 displays the most complex splicing pattern among VGSCs, producing a spectrum of splice variants implicated in cardiac physiology, pathology and cancer biology. One of the earliest identified variants is Nav1.5a (Δexon18), which lacks the 53-amino acid segment encoded by exon 18 within the II–III intracellular linker [38]. Expressed in the heart, brain and dorsal root ganglia, Nav1.5a shows current density comparable to the full-length channel but exhibits distinct inactivation and recovery kinetics, indicating a role in modulating channel gating [39,40].

Further diversity arises from variants such as Nav1.5b and Nav1.5d, which involve partial or complete skipping of exon 17 or inclusion of a truncated exon 18. Nav1.5b forms a non-functional channel; its expression is highly species- and tissue-specific, detectable at very low levels in mouse heart but absent in human or porcine hearts. In contrast to the non-functional Nav1.5b splice variant, which displays highly restricted expression in rodents, Camacho et al. identifies Nav1.5d as a functional and human-specific isoform. This variant is characterized by the in-frame deletion of 40 amino acids within the DII/DIII intracellular linker, resulting in the loss of a putative amphipathic helix and a potential protein kinase A (PKA) phosphorylation site. Nav1.5d is constitutively expressed in human hearts at both embryonic and adult stages, independent of age or sex, accounting for approximately 2–3% of total cardiac *SCN5A* transcripts. Functionally, it generates voltage-gated Na^+^ currents with a markedly reduced amplitude, largely attributable to a decreased single-channel open probability, and exhibits depolarizing shifts in both steady-state activation and inactivation. Its trafficking to the plasma membrane appears normal, and its modulation by PKA remains intact. Structure–function analysis implicates a short motif within the deleted region as a critical determinant of channel gating. Thus, Nav1.5d represents a persistent, functionally distinct human splice variant that may contribute to the fine-tuning of cardiac excitability, particularly within the conduction system, highlighting the species-specific complexity of Nav1.5 regulation [41,42]. The existence of such non-functional isoforms suggests a regulatory mechanism whereby unproductive splicing fine-tunes the abundance of functional channels. Another significant isoform, Nav1.5c (Q1077), is defined by an additional CAG codon in exon 18, inserting a glutamine at position 1077. As a major isoform in the human heart, its functional consequences are under active investigation. A consistent transcript ratio of Nav1.5 to Nav1.5c (∼2:1) is maintained across different cardiac regions [41,43,44].

A critical developmental switch involves the mutually exclusive selection of exon 6 (adult) and exon 6A (neonatal), which encode 30 amino acids differing at seven positions within the domain I S3–S4 linker. The resulting neonatal isoform, Nav1.5e, is re-expressed in breast cancer and other carcinomas, where it promotes invasive behaviour [45,46]. Nav1.5e promotes cancer cell invasion primarily through persistent sodium influx. Its channel activity facilitates the formation of a functional complex with the sodium hydrogen exchanger 1 (NHE1) at invadopodia. This coupling activates NHE1, leading to intracellular alkalinization that promotes protease mediated ECM degradation, while concurrently driving local osmotic swelling and pseudopod extension – collectively enhancing migratory and invasive behaviour. Notably, a specific antibody targeting nNav1.5 effectively blocks its channel activity and suppresses invasion, underscoring its functional role and therapeutic relevance in metastasis. This re-emergent foetal electrophysiological signature is proposed to confer a proliferative or pro-invasive advantage to cancer cells, linking *SCN5A* alternative splicing to oncogenic processes.

### 3.4. Nav1.7: splicing as a modulator of pain pathways

As a key channel in nociception, Nav1.7 undergoes developmentally regulated alternative splicing at two primary loci that refine its functional properties within pain pathways. The first event involves mutually exclusive exons 6N and 6A in domain I, analogous to the splicing switch in central nervous system sodium channels. The 6A (adult) variant significantly slows inactivation kinetics at subthreshold membrane potentials, potentially facilitating action potential initiation during sustained or slow depolarizing stimuli [47]. The second event occurs in exon 12, generating isoforms that either include (12L) or lack (12S) an 11-amino acid segment within the intracellular I–II linker [47,48]. While these variants exhibit similar baseline biophysical properties, the 12S isoform is uniquely susceptible to modulation by PKA. Specifically, cAMP-induced phosphorylation induces a hyperpolarizing shift in the voltage dependence of activation for 12S-containing channels, thereby lowering the threshold for action potential generation [48]. This coupling between a specific splice variant and an intracellular second-messenger pathway constitutes a potent mechanism for fine-tuning neuronal excitability and pain sensitivity. Notably, peripheral nerve injury alters the relative abundance of Nav1.7 splice variants in dorsal root ganglia, implicating regulated splicing in the pathogenesis of neuropathic pain [49].

### 3.5. Peripheral nervous system-specific splicing: Nav1.8 and Nav1.9

Alternative splicing further diversifies VGSCs in the peripheral nervous system, with Nav1.8 and Nav1.9 exhibiting distinct splicing mechanisms. In *SCN10A* (Nav1.8), a subtle splicing event at a NAGNAG tandem acceptor site in exon 17 generates isoforms that either include or exclude a single glutamine residue at position 1030 within the II–III intracellular linker. Although phylogenetically conserved across humans, rats and mice, the resulting isoform ratios display marked species specificity: the − Q variant predominates in human and rat dorsal root ganglia, whereas the + Q variant is most abundant in mice. Biophysical analyses indicate this splice choice does not significantly alter channel gating or kinetics in humans, suggesting it may represent a neutral evolutionary event or involve functional roles yet to be determined [50]. In contrast, *SCN11A* (Nav1.9) undergoes alternative splicing that produces two principal isoforms: the full-length Nav1.9a and Nav1.9b, the latter incorporating a novel cassette exon (E23c) derived from an intronic sequence. Inclusion of E23c introduces a frameshift predicted to generate a truncated, non-functional protein. Utilization of E23c is suppressed in humans and mice but occurs in rats, illustrating how species-specific regulation of splicing and *de novo* exon creation via intron-to-exon conversion can contribute to functional diversification – even when the immediate outcome is a loss-of-function isoform [51].

### 3.6. A pattern analysis of VGSC alternative splicing

A systematic comparison of the alternative splicing events across the aforementioned VGSC subtypes (Nav1.1–Nav1.9) reveals two predominant regulatory patterns that collectively shape the functional diversity of this channel family: deeply evolutionarily conserved core splicing switches, and diverse splicing events driving specific functional adaptations.

A prominent manifestation of evolutionary conservation is the mutually exclusive exon selection occurring at corresponding positions in the first (DI) and third (DIII) domains. In the S3–S4 linker of the voltage-sensing domain in DI, homologous structural splicing switches exist in Nav1.1 (exon 5N/5A), Nav1.2 and Nav1.3 (exon 6N/6A), Nav1.5 (exon 6N/6A), Nav1.6 (exon 5N/5A) and Nav1.7 (exon 5N/5A). The regulation of these switches is crucial for the developmental maturation of voltage dependence and tissue-specific adaptation of the channels [52,53]. Of greater evolutionary significance is the presence of an analogous pair of mutually exclusive exons, 18N/18A, in DIII of Nav1.6. The conserved splicing patterns at structurally corresponding positions in both DI and DIII provide key supporting evidence for the hypothesis that the VGSC α subunit originated from a ‘two-domain ancestral’ channel. Following gene duplication of this ancestor, the primordial splicing regulatory module was symmetrically retained within the two homologous domains produced by the duplication, serving as an evolutionary signature preserved throughout the gene family [54].

In stark contrast, another category of splicing events exhibits high subtype-, species- or developmental stage-specificity, serving as a primary mechanism for functional fine-tuning and environmental adaptation. For instance, the Nav1.5 splice variants Nav1.5b (mouse-specific, non-functional) and Nav1.5d (human-specific, with altered function) reveal the splicing basis for electrophysiological differences between species [41]; the distinct expression profiles of *SCN8A* exon 18 variants in neuronal versus non-neuronal cells (e.g. inner ear hair cells) precisely match the specific excitability requirements of these cell types [55]; and the developmental switch from ‘neonatal’ to ‘adult’ isoforms across various channels exemplifies the precise temporal regulation of splicing during nervous system maturation. Therefore, the landscape of VGSC alternative splicing is shaped by a deeply conserved framework rooted in its evolutionary history, coexisting with highly plastic peripheral events that have arisen to meet specific physiological demands. Understanding the interplay between these two modes is key to elucidating the functional diversity of VGSCs and their central role in development, plasticity and disease.

### 3.7. VGSC alternative splicing and cancer

VGSCs are widely dysregulated in various cancers (such as breast, prostate, cervical and colon cancers), with different cancer types preferentially expressing specific α-subunits. For instance, Na_v_1.5 is dominant in breast cancer, while Na_v_1.7 is predominant in prostate cancer. The expression of these channels is closely associated with aggressive tumour phenotypes, promoting cancer cell migration, invasion, endocytosis, secretion and related behaviours. Aberrant splicing of VGSC subtypes plays a key role in cancer progression, particularly in breast and prostate cancers. In these cancers, the α-subunits (e.g. Na_v_1.5 and Na_v_1.7) often generate neonatal splice variants, which exhibit electrophysiological characteristics such as slowed channel inactivation and enhanced persistent sodium current, thereby significantly increasing the migratory and invasive capacities of cancer cells. In contrast, adult splice isoforms are primarily expressed in colon cancer [56].

At the molecular mechanism level, aberrantly spliced VGSC isoforms, particularly the neonatal Na_v_1.5 highly expressed in breast cancer, synergistically drive tumour progression through multiple interconnected mechanisms. Due to splicing alterations, these isoforms exhibit enhanced persistent sodium current and slowed inactivation among other electrophysiological properties. The sodium influx mediated by these channels, on the one hand, activates the sodium-hydrogen exchanger (NHE-1), leading to intracellular alkalinization and localized extracellular acidification, which in turn activates acid-dependent proteases such as cathepsins to degrade the ECM. On the other hand, sodium influx activates Src family kinases, which phosphorylate cortactin, promoting actin polymerization and the formation/stabilization of invasive protrusions (invadopodia). Meanwhile, VGSCs co-localize with NHE-1 in caveolin-1-enriched lipid rafts, further cooperatively enhancing the ECM-degrading function of invadopodia. Furthermore, VGSC activity promotes endocytosis and exosomal vesicle trafficking, accelerating protease secretion and matrix remodelling, and modulates invasion-related gene networks to influence cancer cell behaviour, while extracellular vesicles may be potential targets for reversing chemotherapy resistance [57]. Notably, in certain cell types, VGSCs (e.g. Naáµ¥1.5) located on late endosome membranes regulate endosomal acidification, affecting vesicle trafficking and microenvironment remodelling. These electrophysiological, biochemical, structural and genetic alterations interact to collectively establish a multi-layered pro-invasive signalling platform, thereby significantly enhancing the migratory, locally invasive and metastatic capabilities of cancer cells [58].

VGSC isoforms generated by aberrant splicing exhibit absent or extremely low expression in normal adult tissues but are specifically highly expressed in tumours, endowing them with the potential to serve as ideal biomarkers. For instance, the re-expressed ‘neonatal’ Nav1.5 (Nav1.5e) isoform in breast cancer can function as a diagnostic marker to distinguish tumour tissue from normal tissue [59]. With the advancement of liquid biopsy technologies, detecting the mRNA or protein products of such cancer-specific VGSC splice variants in circulating tumour cells (CTCs) or exosomes provides a novel approach for minimally invasive, dynamic disease monitoring and treatment response evaluation. Furthermore, aberrant splicing itself is a significant source of tumour neoantigens. When splicing events – such as exon skipping, mutually exclusive exon selection, or intron retention – generate novel exon–exon junctions not present in the normal genome, they translate into unique ‘frameshift’ or *de novo* peptide sequences. These splicing-derived neoantigens can be recognized by the immune system as ‘non-self,’ thereby stimulating anti-tumour T-cell responses. The complex splicing patterns of VGSC genes make them a rich reservoir for such neoantigens. Recent advances in computational immunology, including bioinformatics tools like pVACsplice, now enable the systematic prediction and prioritization of neoantigens arising from aberrant splicing based on tumour RNA sequencing data [60]. By analysing the splicing landscape of VGSC genes, it is possible to identify neoantigenic peptides encoded by cancer-specific splicing events that exhibit high-affinity binding to the patient’s individual HLA molecules, which can subsequently be utilized in the design of mRNA vaccines.

## 4. Regulation of alternative splicing in voltage-gated sodium channels

The precise patterns of VGSC alternative splicing are orchestrated by a multi-layered regulatory network. This system integrates intrinsic genetic signals with extrinsic physiological cues to direct the inclusion or skipping of specific exons in a cell-type-specific, developmentally programmed and activity-modulated manner. Central to this process are RBPs, which recognize cis-regulatory elements within pre-mRNA transcripts to catalyse spliceosome assembly and determine splicing outcomes [61]. Deciphering this combinatorial regulatory code is essential for understanding VGSC biology and for developing future strategies to correct splicing defects associated with channelopathies.

### 4.1. The central role of the Rbfox family in neuronal splicing control

The Rbfox family of RBPs (Rbfox1/2/3) functions as a master regulator of alternative splicing in neurons, profoundly influencing VGSC transcript processing. These proteins contain a highly conserved RNA recognition motif that specifically binds (U)GCAUG motifs, typically located in intronic regions flanking alternative exons. A canonical example of their precision is the regulation of *SCN8A* (Nav1.6) splicing, where Rbfox1 and Rbfox2 directly promote inclusion of the critical exon 18A, which is essential for producing full-length functional channels [35,62]. This activation is mediated through binding to conserved UGCAUG elements downstream of exon 18A, facilitating the recruitment of the core spliceosomal machinery. The developmental upregulation of Rbfox proteins coincides with the switch from the non-functional 18N to the functional 18A isoform, and genetic ablation studies substantiate this essential role: Rbfox1^+/−^; Rbfox2^−/−^ double heterozygous mice show a severe reduction in exon 18A inclusion, accompanied by decreased Nav1.6 protein expression and prominent neurological deficits, including impaired pacemaking activity in Purkinje neurons. Furthermore, Rbfox proteins coordinate the developmental switching of domain I exons (5A/5N) in *SCN8A*, illustrating multi-layered control over the channel’s biophysical properties. The regulatory scope of Rbfox extends beyond *SCN8A*, as its characteristic binding motifs are enriched near alternative exons of several other VGSC genes, suggesting a conserved splicing control mechanism across the channel family [25]. Notably, Rbfox expression in non-neuronal cell types, such as oligodendrocyte precursor cells and cardiac myocytes, correlates with the presence of functional sodium currents, indicating a potential role in shaping the electrical phenotype of these cells through splicing-mediated channel repertoire modulation.

### 4.2. Splicing modifiers with channel- and exon-specific functions

Beyond broadly acting regulators like the Rbfox family, VGSC alternative splicing is further refined by additional RBPs and core spliceosomal components that confer channel- and exon-specific regulation. A key example is sodium channel modifier 1 (SCNM1), implicated in splicing multiple VGSC transcripts. SCNM1 physically interacts with the spliceosome and modulates its fidelity and substrate specificity. A mutation in SCNM1 exacerbates disease severity in a mouse epilepsy model, likely through altered *SCN8A* splicing. Recent evidence links SCNM1 to regulating the neonatal exon 5N in SCN1A, where its dysfunction may promote epileptogenesis by shifting the 5A/5N ratio, positioning SCNM1 as a core spliceosomal component with targeted effects on critical VGSC exons [63,64]. Another regulator is the neuron-specific RBP Nova2, known for synaptic RNA processing. In human *SCN1A*, Nova2 promotes inclusion of the neonatal exon 5N. Bioinformatic and experimental analyses indicate its activity is highly exon-specific, primarily targeting the exon 5 region of *SCN3A* and potentially *SCN1A*, but not significantly influencing *SCN2A* or *SCN8A* under physiological conditions [30,65,66]. This highlights the precision of the splicing code, whereby distinct RBPs are selectively deployed to fine-tune individual alternative exons within specific VGSC genes, adding a critical layer of regulatory specificity to neuronal channel function.

### 4.3. Integrated splicing networks and future directions

The regulation of VGSC alternative splicing operates through integrated networks, where Rbfox, Nova2, SCNM1 and other factors (including SR (serine/arginine) and hnRNP proteins) engage in competitive, cooperative or antagonistic interactions on shared pre-mRNA substrates. The ultimate splicing output is determined by the combinatorial activity of these trans-acting factors, whose functions are further modulated by neuronal activity, cellular stress and developmental signalling pathways. For example, neuronal activity can influence splicing through calcium-dependent signalling cascades. This pathway modifies the phosphorylation status of splicing regulatory factors (such as SR proteins), thereby altering their RNA-binding affinity or subcellular localization [67]. Developmental signalling pathways strictly regulate isoform switching, as seen with the neonatal splice form of Nav1.5 (nNav1.5), which is highly expressed in the developing heart and brain but rapidly downregulated in adulthood – a process likely coordinated by developmental signals [68]. Additionally, the function of splicing factors themselves can be regulated by genetic variants. For instance, mutations in the splicing factor SCNM1 can act as genetic modifiers that exacerbate disease severity in mice by reducing the splicing accuracy at mutated donor sites [64].

## 5. Therapeutic targeting of voltage-gated sodium channelopathies: from broad blockers to precision splicing correction

VGSCs are critical therapeutic targets for a spectrum of channelopathies due to their fundamental role in electrical excitability [69]. Historically, clinical management has relied on small-molecule agents – such as local anaesthetics, antiarrhythmic drugs and classical antiepileptics – that function primarily as state-dependent sodium channel blockers [70,71]. These compounds typically stabilize the inactivated channel state, reducing the available channel pool to attenuate pathological hyperexcitability. However, their limited subunit selectivity often leads to substantial off-target effects and suboptimal efficacy, particularly in monogenic channelopathies [72]. Advances in understanding VGSC genetics and molecular pathophysiology, including the regulatory role of alternative splicing, are now enabling a shift towards precision medicine [73]. This section examines the evolution of VGSC-directed therapeutics, focusing on emerging strategies that aim to directly modulate the splicing machinery to achieve isoform-specific correction [74].

### 5.1. The limitations and promise of conventional sodium channel blockers

Conventional sodium channel blockers – including phenytoin, carbamazepine and lamotrigine – remain first‑line therapies for epilepsy and neuropathic pain, affirming the therapeutic significance of VGSCs. Their mechanism, however, involves binding to conserved structural regions such as the inactivation gate or pore domain, which are shared across most VGSC subtypes. This results in non‑selective inhibition of all channels within affected tissues and explains the frequent dose‑limiting adverse effects – such as dizziness, ataxia and cognitive impairment – primarily due to blockade of key central nervous system isoforms like Nav1.1, Nav1.2 and Nav1.6 [75,76]. Clinical efficacy is further modulated by the expression of specific splice variants. For example, the neonatal Nav1.1 isoform containing exon 5N confers resistance to carbamazepine compared with the adult 5A variant, providing a mechanistic explanation for differential drug responses in patients with *SCN1A*‑related epilepsies [77,78]. These observations highlight that individual splicing profiles must be considered for the rational selection of sodium channel‑targeted therapeutics.

### 5.2. Antisense oligonucleotides: a modular platform for precision therapy

Antisense oligonucleotides (ASOs) are synthetic single‑stranded nucleic acids that achieve high specificity via Watson–Crick base pairing with complementary RNA sequences. Their modular design allows precise targeting of nearly any RNA transcript, offering a powerful alternative to conventional small‑molecule inhibitors [79]. ASOs modulate gene expression through several mechanisms, two of which are especially relevant for VGSC channelopathies. First, in the RNase H1‑mediated degradation pathway, ASOs hybridize to target mRNA and recruit endogenous ribonuclease H1, leading to transcript cleavage and reduced protein levels – a strategy suited for disorders driven by gain‑of‑function mutations. Second, ASOs can act as splicing modulators by binding pre‑mRNA and sterically blocking access of splicing regulators to key cis‑elements (e.g. splice sites, exonic or ISEs), thereby redirecting spliceosome assembly to alter exon inclusion [80,81]. This approach can be used to skip mutant exons, restore productive reading frames or fine‑tune the stoichiometry of physiological isoforms, offering a rational means to correct disease‑associated splicing defects in VGSC genes.

### 5.3. ASO success stories in preclinical models of VGSC disorders

ASOs have shown therapeutic promise in preclinical models of severe genetic epilepsies, demonstrating both transcript degradation and splicing modulation as viable strategies for VGSC-related disorders. In Dravet syndrome – primarily caused by *SCN1A* haploinsufficiency and a consequent deficit in functional Nav1.1 protein – the TANGO (targeted augmentation of nuclear gene output) approach was developed to counteract non-productive *SCN1A* pre-mRNA splicing [82]. This ASO binds to a cryptic exon whose inclusion introduces a premature termination codon, triggering NMD. By blocking this aberrant splicing, the ASO promotes productive, full-length *SCN1A* mRNA. In a Dravet mouse model, this intervention restored physiological Nav1.1 protein levels, substantially reduced seizure frequency, and lowered the incidence of sudden unexpected death in epilepsy (SUDEP), illustrating a paradigm-shifting strategy for loss-of-function channelopathies [83]. Conversely, for encephalopathies resulting from gain-of-function mutations in *SCN2A* or *SCN8A*, ASOs have been used to selectively suppress mutant allele expression. In a mouse model carrying the *Scn2a* R1882Q epileptogenic mutation, a *Scn2a*-directed ASO reduced mutant transcript levels, prevented premature lethality and suppressed spontaneous seizures, with treated animals exhibiting normal behaviour [84]. Similarly, in an *SCN8A*-related encephalopathy model, neonatal delivery of an ASO that induces RNase H1-dependent degradation of Scn8a mRNA significantly extended survival and attenuated seizure burden, underscoring the efficacy of early suppression of toxic channel isoforms in hyperexcitable disorders [85,86]. Together, these successes highlight the versatility of ASOs in addressing diverse molecular pathologies within the VGSC family.

### 5.4. Limitations in the clinical translation of ASOs

However, ASOs face two major limitations in the process of clinical translation. The first is off-target effects. ASOs are typically designed to be 15–25 nucleotides in length and are theoretically highly sequence-specific. However, their binding targets are often conserved sequences near splice sites, which paradoxically increases the likelihood of off-target interactions. Research by Scharner et al. investigated off-target effects of splice-modulating ASOs *in vitro*: among 108 potential off-target sites predicted based on sequence complementarity, 17 mis-splicing events were confirmed [87]. Off-target effects of ASOs may lead to unpredictable side effects, making it crucial to conduct comprehensive and systematic cytotoxicity and individual toxicity assessments for candidate ASOs.

The other major limitation lies in the targeting and delivery efficiency of ASOs. Taking the success of nusinersen as an example, it provides an important paradigm for targeting the central nervous system via intrathecal administration to treat epileptic encephalopathies related to VGSCs [88]. However, achieving targeted delivery to peripheral tissues through subcutaneous injection or intravenous infusion is considerably more complex. On one hand, the uptake efficiency of ASOs varies significantly among different peripheral tissues; for instance, the liver exhibits much higher uptake of nusinersen compared to the kidneys [89]. On the other hand, systemically delivered ASOs typically lack tissue specificity, which greatly restricts the application of VGSC-targeting ASOs in areas such as anti-cancer therapy or analgesia. To address this issue, tissue-specific modifications of ASOs represent one of the current effective strategies. For example, ASOs conjugated with GalNAc3 can be specifically taken up by the liver [90].

### 5.5. Beyond ASOs: emerging therapeutic avenues

While ASOs represent a leading RNA-targeted therapeutic approach, advances in understanding splicing regulation have spurred the development of additional strategies. Small-molecule splicing modulators, exemplified by the clinical success of risdiplam and branaplam in spinal muscular atrophy, constitute a promising class of therapeutics [91,92]. These compounds typically bind specific structural motifs in pre‑mRNA or associated splicing factor complexes to promote inclusion of a target exon – such as SMN2 exon 7. This paradigm could, in principle, be extended to voltage-gated sodium channelopathies; for instance, through orally available small molecules that enhance inclusion of the adult 5A exon in *SCN1A* or induce skipping of pathogenic exons in other VGSC genes. In parallel, CRISPR-based splicing correction systems offer a fundamentally distinct therapeutic avenue. By employing a catalytically inactive Cas9 (dCas9) fused to effector domains that function as splicing activators or repressors, this technology allows precise genomic targeting to directly reprogram splicing outcomes [93]. Such an approach holds potential for durable, single-intervention correction of aberrant splicing patterns, positioning CRISPR–dCas9 systems as a next-generation strategy for permanently treating splicing-related channelopathies.

## 6. Conclusions and future perspectives

Research on VGSCs has established that their functional diversity and pathological roles extend far beyond the existence of nine distinct genes. As comprehensively described in this review, alternative splicing serves as a fundamental and widespread mechanism that post-transcriptionally expands the proteomic and functional landscape of the VGSC family. The complex splicing profiles of all nine Nav α subunits reveal intricate patterns of developmentally regulated switches, tissue-specific isoforms and biophysical fine-tuning. By integrating the current understanding of VGSC alternative splicing, this review provides a framework to catalyse these efforts and accelerate the development of precise and effective therapies for severe, refractory neurological channelopathies and certain cancers.

Several overarching principles emerge. First, the conserved mutually exclusive splicing of exons 5A/5N in the first domain of neuronal channels (e.g. Nav1.1, Nav1.2, Nav1.6) constitutes a precisely regulated node with direct clinical consequences, affecting drug responses and the severity of early-onset epilepsies. Second, the regulation of these events by specific RBPs (e.g. Rbfox and Nova2) reveals a sophisticated ‘splicing code’ that integrates genetic programs with cellular functional demands. Third, the dysregulation of this machinery – whether through *cis*-element mutations or dysfunction of *trans*-acting factors – defines a distinct class of ‘splicing channelopathies,’ in which an imbalance of the splice variants themselves drives the disease.

The majority of splicing isoform switching in VGSCs is context- or time-dependent, exemplified by the 5N and 5A isoforms of *SCN5A*. It is likely that each VGSC undergoes alternative splicing at analogous positions, but in a temporally and spatially specific manner. According to the context-dependent mechanism of alternative splicing regulation, the final alternative splicing pattern is determined by the collective action of numerous RBPs, extending beyond the specific examples such as RBFOX, SCNM1 and NOVA mentioned previously. Therefore, future systematic investigation of RBPs with binding potential to VGSC pre-mRNAs is necessary to construct the regulatory network governing VGSC alternative splicing. Methods such as ChIRP-MS, RAP-MS and CARPID are viable options for this purpose [94]. Beyond expanding the catalog of known VGSC splicing isoforms, this knowledge would also provide crucial insights for the precise, targeted modulation of VGSCs.

## Data Availability

Data availability is not applicable to this article as no new data were created or analysed in this study.
